# Molecular mechanisms of *Streptococcus pyogenes* Cas9: a single-molecule perspective

**DOI:** 10.52601/bpr.2021.210021

**Published:** 2021-12-31

**Authors:** Qian Zhang, Ziting Chen, Bo Sun

**Affiliations:** 1 School of Life Science and Technology, ShanghaiTech University, Shanghai 201210, China; 2 CAS Center for Excellence in Molecular Cell Science, Shanghai Institute of Biochemistry and Cell Biology, Chinese Academy of Sciences, Shanghai 200031, China; 3 University of Chinese Academy of Sciences, Beijing 100049, China; 4 School of Chemistry and Chemical Engineering, Frontiers Science Center for Transformative Molecules and National Center for Translational Medicine, Shanghai Jiao Tong University, Shanghai 200240, China; 5 Gene Editing Center, ShanghaiTech University, Shanghai 201210, China

**Keywords:** CRISPR, Cas9, Single-molecule, Molecular mechanism, Nuclease, R-loop

## Abstract

Cas9 is an RNA-guided endonuclease from the type II CRISPR-Cas system that employs RNA–DNA base pairing to target and cleave foreign DNA in bacteria. Due to its robust and programmable activity, Cas9 has been repurposed as a revolutionary technology for wide-ranging biological and medical applications. A comprehensive understanding of Cas9 mechanisms at the molecular level would aid in its better usage as a genome tool. Over the past few years, single-molecule techniques, such as fluorescence resonance energy transfer, DNA curtains, magnetic tweezers, and optical tweezers, have been extensively applied to characterize the detailed molecular mechanisms of Cas9 proteins. These techniques allow researchers to monitor molecular dynamics and conformational changes, probe essential DNA–protein interactions, detect intermediate states, and distinguish heterogeneity along the reaction pathway, thus providing enriched functional and mechanistic perspectives. This review outlines the single-molecule techniques that have been utilized for the investigation of Cas9 proteins and discusses insights into the mechanisms of the widely used* Streptococcus pyogenes* (Sp) Cas9 revealed through these techniques.

## INTRODUCTION

Clustered regularly interspaced short palindromic repeats (CRISPR) and CRISPR-associated (Cas) proteins are widely found in bacterial and archaeal genomes as adaptive immune systems that offer defense against attacks from foreign genetic elements, such as viruses and plasmids (Barrangou* et al.* 2007; Brouns* et al.* 2008; Marraffini and Sontheimer 2008, 2010). The CRISPR–Cas immune response consists of three main stages. Once invasive genetic elements are detected, Cas proteins first split the invading foreign DNA into small pieces and integrate these fragments into the CRISPR loci region. This process is referred to as adaptation, which gives rise to the formation of genetic memory of invading nucleic acids (Heler* et al.* 2015; Silas* et al.* 2016; Sternberg* et al.* 2016; Wei* et al.* 2015). Subsequently, transcription of the newly formed CRISPR loci region generates precursor CRISPR RNAs (pre-crRNAs), which are further processed into mature crRNAs (crRNA biogenesis) that can be associated with Cas proteins to form effector complexes (Carte* et al.* 2008; Charpentier* et al.* 2015; Deltcheva* et al.* 2011; Liu* et al.* 2017; Staals* et al.* 2013, 2014; Zhang* et al.* 2013b). At the last interference stage, the effector complexes locate DNA targets complementary to their crRNAs and carry out the degradation of invading nucleic acids (Jinek* et al.* 2012; Jore* et al.* 2011).

Recent years have witnessed a substantial increase in the diversity of CRISPR–Cas systems. To date, CRISPR–Cas systems have been classified into two classes, six types, and 33 subtypes (Makarova* et al.* 2020). The major difference between Class I and II CRISPR–Cas systems is the composition of the effector modules. The effectors of the Class I systems are composed of multiple Cas proteins, while the Class II CRISPR–Cas systems use a single protein that functions similarly to the entire effector complex of Class I (Hayes* et al.* 2016; Jinek* et al.* 2012; Koonin* et al.* 2017). Based on the presence of the signature Cas proteins, crRNA processing, and target recognition, the two classes are further classified into various types and subtypes. We refer readers to a comprehensive review for more details on the classification of the CRISPR–Cas systems (Makarova* et al.* 2020).

The Cas9 protein is the sole nuclease in the effector complex from Type II of the Class II CRISPR system (Barrangou* et al.* 2007; Garneau* et al.* 2010; Sapranauskas* et al.* 2011). A single Cas9 endonuclease complexed with a dual guide RNA (gRNA) comprising crRNA and *trans*-activating crRNA (tracrRNA) is sufficient to target and cleave complementary ~20-base-pair (bp) DNA sequences that have a short protospacer adjacent motif (PAM) located immediately downstream of the sequences (Deltcheva* et al.* 2011; Garneau* et al.* 2010; Gasiunas* et al.* 2012; Jinek* et al.* 2012; Karvelis* et al.* 2013). The system can be further simplified by fusing the two RNA molecules into a single guide RNA (sgRNA) (Jinek* et al.* 2012). Due to its simplicity and programmability, the Cas9 protein has been widely repurposed as an effective RNA-guided DNA-targeting platform that can easily modify the genome in various species (Knott and Doudna 2018; Zhang 2019). In addition, the nuclease-deficient version of the enzyme (dCas9) has also been widely used in transcriptional regulation and *in vivo* imaging (Bikard* et al.* 2013; Chen* et al.* 2013; Gilbert* et al.* 2013, 2014; Hilton* et al.* 2015; Konermann* et al.* 2013, 2015; Ma* et al.* 2015, 2016b; Maeder* et al.* 2013; Perez-Pinera* et al.* 2013; Qi* et al.* 2013; Tanenbaum* et al.* 2014; Thakore* et al.* 2015). To ensure their efficiency and accuracy, a thorough understanding of the molecular mechanisms of Cas9 proteins would aid in increasing their fidelity and minimizing off-target effects.

The functional nuclease activity of Cas9–sgRNA complexes commonly requires PAM search and recognition, protospacer DNA unwinding, R-loop formation, and subdomain conformational rearrangement (Anders* et al.* 2014; Jiang* et al.* 2015, 2016b; Sternberg* et al.* 2014, 2015). Although ensemble studies have contributed enormously to the understanding of the molecular mechanisms of Cas9 proteins, these ensemble approaches often reveal the average molecular population and lack the ability to detect intermediate states and distinguish heterogeneity of Cas9 proteins. In the past decade, single-molecule techniques have become the complementary approaches that help understand the detailed molecular dynamics of Cas9 proteins (Cuculis and Schroeder 2017; Globyte* et al.* 2018; Singh and Ha 2018; Whinn* et al.* 2019). These techniques offer the ability to provide enriched information on each specific step of Cas9 proteins along its pathway towards catalysis. Moreover, single-molecule manipulation techniques, such as optical tweezers and magnetic tweezers, offer the capability to apply external mechanical force and torque on DNA substrates (Mullally* et al.* 2020; Newton* et al.* 2019; Szczelkun* et al.* 2014; Zhang* et al.* 2019). Using these techniques, a more comprehensive understanding of the Cas9 mechanism can be generated. In this review, we introduce the major single-molecule approaches used to study Cas9 proteins and highlight examples of insights into the molecular mechanisms of the best-characterized *Streptococcus pyogenes* (Sp) Cas9 obtained using these methods.

## SINGLE-MOLECULE TECHNIQUES FOR Cas9 STUDIES

Compared with ensemble studies, single-molecule approaches have shown great advantages in measurements of molecular heterogeneity, distributions in molecular behaviors, and real-time dynamics of single biomolecules (Cordes* et al.* 2015; Cuculis and Schroeder 2017; Leake 2013; Sun 2019; Sun and Wang 2016). A wide variety of fluorescence spectroscopy-based (DNA curtains and fluorescence resonance energy transfer) and force spectroscopy-based (magnetic tweezers, optical tweezers, and atomic force microscopy) single-molecule techniques have been used to investigate different aspects of Cas9 proteins (Cuculis and Schroeder 2017; Globyte* et al.* 2018; Singh and Ha 2018; Whinn* et al.* 2019). In this section, we will briefly describe their principles and capabilities.

### DNA curtains

**Figure 1:**
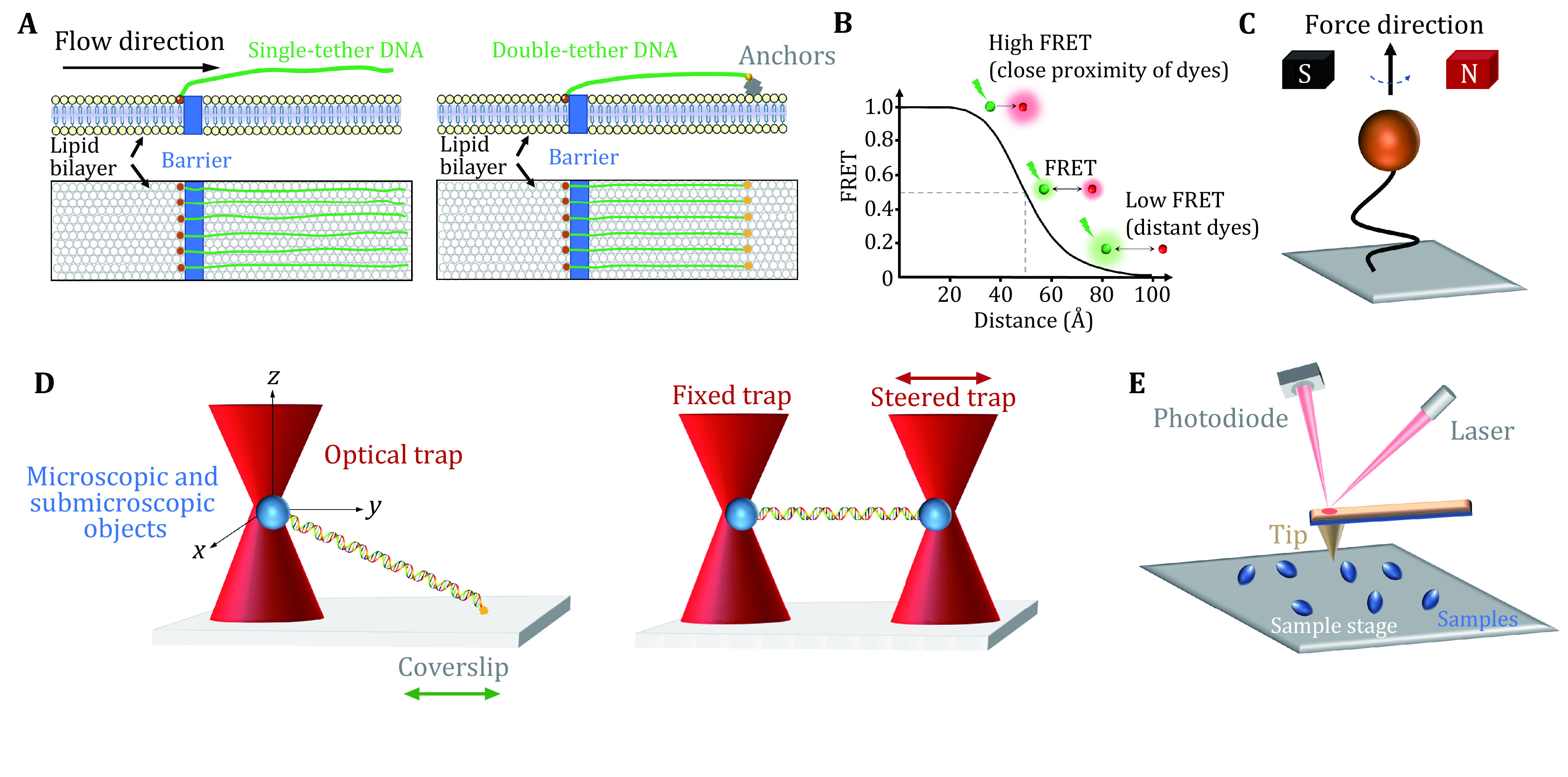
Single-molecule techniques for the study of Cas9 proteins. **A** DNA curtains. Single-tethered DNA curtains (left). An array of DNA molecules is aligned by a barrier in a lipid bilayer, while laminar flow stretches the molecules away from the barrier. Double-tethered DNA curtains (right). Two ends of a DNA molecule are respectively fixed to the barrier and the anchors (yellow) on the phospholipid bilayer. **B** smFRET. Schematic of the FRET efficiency as a function of the distance (*R*) between a pair of dyes for *R*_0_ = 50 Å. The donor dye transfers energy to the acceptor dye. The transfer efficiency depends on the distance between the two dyes. **C** Magnetic tweezers. One end of a double-stranded DNA molecule is usually attached to the glass surface, and the other end is attached to a magnetic bead. The magnetic field can apply force and torque to the magnetic bead, thereby manipulating the DNA molecules. **D** Optical tweezers. A highly focused laser beam can capture and move microscopic and submicroscopic objects, such as polystyrene beads, thereby manipulating and monitoring the DNA molecule attached to them. Single optical tweezers (left) typically require the ends of a DNA molecule attached to a bead and the coverslip surface. In a dual optical tweezers (right) configuration, two ends of a DNA molecule are attached to two beads manipulated by two traps. **E** AFM. The tip of the needle fluctuates in the direction perpendicular to the surface of the sample under the action of a constant repulsive force so that information on the surface morphology of the sample can be obtained

DNA curtains refer to a methodology that aligns hundreds of DNA molecules on the surface of a microfluidic sample chamber wherein fluorescently labeled DNA and proteins can be monitored simultaneously under total internal reflection fluorescence (TIRF) microscopy (Axelrod 1989; Fazio* et al.* 2008; Greene* et al.* 2010). This approach commonly uses biotin and streptavidin in the phospholipid bilayer on a quartz wafer to anchor multiple DNA molecules (Fig. 1A, left). Due to the barriers in the phospholipid bilayer, one end of DNA molecules is fixed and aligned, and the others, driven by flow, are free-floating (Fazio* et al.* 2008). Alternatively, to avoid the waste of samples and the perturbation from the flow, both ends of a relatively stretched DNA molecule can be anchored (Fig. 1A, right) (Gorman* et al.* 2010). In this scenario, the flow is not needed. DNA curtains are a high-throughput technique that allows real-time visualization of hundreds of fluorescently labeled DNA and proteins. This technique has been successfully applied to investigate PAM search, DNA association and dissociation of SpCas9 (Table 1) (Cuculis* et al.* 2016; Redding* et al.* 2015; Sternberg* et al.* 2014).

**Table 1:** Single-molecule approaches applied to investigate each catalytic step of Cas9

Cas9 activity	Single-molecule methods
PAM search	DNA curtains, FRET
DNA target binding	DNA curtains, FRET, Optical tweezers, AFM
Protospacer unwinding and R-loop formation	Magnetic tweezers, FRET
Conformational rearrangement	FRET, AFM
Dissociation from DNA	DNA curtains, FRET, Optical tweezers

### Fluorescence resonance energy transfer

Single-molecule fluorescence resonance energy transfer (smFRET) is a powerful technique that can monitor nanometer-scale change in biological macromolecules, such as DNA and protein. By measuring the resonance energy transfer efficiency (*E*) between a donor and an acceptor dye, this approach reveals the distance change between the labeling sites in real time (Roy* et al.* 2008). smFRET measurements are generally carried out under TIRF microscopy with a light source (usually a laser) irradiating the fluorescently labeled donor molecules on a glass surface. As shown in Fig. 1B, the FRET signals reflect the sensitive change in the distance between the dyes, ranging from 2–8 nm (Roy* et al.* 2008; Selvin 2000). The high spatial and temporal resolution of smFRET allows to detect minute changes and capture transient intermediates between two molecules or within a single molecule. Therefore, smFRET has been involved in the investigation of nearly all catalytic steps of SpCas9 (Table 1) (Bak* et al.* 2021; Chen* et al.* 2017; Dagdas* et al.* 2017; Globyte* et al.* 2019; Lim* et al.* 2016; Okafor* et al.* 2019; Osuka* et al.* 2018; Singh* et al.* 2016, 2018; Sternberg* et al.* 2015; Sung* et al.* 2018; Wang* et al.* 2021; Yang* et al.* 2018, 2021; Zeng* et al.* 2018; Zhang* et al.* 2021).

### Magnetic tweezers

Magnetic tweezers are a single-molecule manipulation technology that can apply force and torque to a group of single DNA molecules (Charvin* et al.* 2005; Gupta* et al.* 2009; Jiang* et al.* 2016a; Sarkar and Rybenkov 2016; Strick* et al.* 1998). In this approach, the two ends of a linear DNA molecule are usually attached to a glass surface and a magnetic bead, respectively (Fig. 1C). A gradient magnetic field exerts force and torque on the magnetic beads, thus manipulating the DNA molecule (Jiang* et al.* 2016a; Strick* et al.* 1996, 1998). By adjusting the external magnetic field, the magnetic beads can be pulled or rotated so that the attached DNA molecules can be stretched or twisted (Cheezum* et al.* 2001; Manosas* et al.* 2010; Sbalzarini and Koumoutsakos 2005). Magnetic tweezers have the advantages of naturally maintaining a constant force within 0.1–100 pN on the DNA and introducing DNA supercoiling under low forces. In the torsionally constrained configuration, magnetic tweezers allow for the sensitive detection of minute changes in DNA length caused by the separation of a few base pairs of dsDNA. Therefore, the R-loop formation induced by SpCas9 proteins can be detected (Table 1) (Mullally* et al.* 2020; Szczelkun* et al.* 2014).

### Optical tweezers

Optical tweezers apply highly focused laser beams to capture and move microscopic and submicroscopic objects (Fig. 1D) (Ashkin* et al.* 1986; Bustamante* et al.* 2021). The single-beam particle trap is generated by an optical radiation pressure gradient force that can flexibly capture samples from several nm to tens of nm, such as biological macromolecular particles (spheres) and organelles (Zhang* et al.* 2013a). The ability of optical tweezers to gently manipulate microscale objects suits the study of “fragile” biological macromolecules. Optical traps can manipulate dielectric microspheres that are attached to biomolecules and detect their positions and forces in real time. This technique offers flexible control of the force and extension of the substrate, thus enabling a quick switch between different modes of operation (Finer* et al.* 1994; Smith* et al.* 1996; Wang* et al.* 1998; Zhang* et al.* 2019, 2020). Optical tweezers provide capabilities to analyze the dynamics of molecules on the spatial scale of a nanometer (nm), the time scale of a millisecond (ms), and the force scale of piconewton (pN, 10^−12^ N) (Maragò* et al.* 2010; Polimeno* et al.* 2018; Zhang* et al.* 2013a).

In single-trap optical tweezers, two ends of DNA molecules are typically attached to the microsphere and the surface of the coverslip, respectively (Fig. 1D, left). Dual optical tweezers suspend a DNA molecule via two traps. This experimental configuration isolates the measurements from the sample chamber, thereby reducing instrument noise (Fig. 1D, right). Critical SpCas9–DNA interactions and the force impact on SpCas9 binding were reported via the use of optical tweezers (Table 1) (Newton* et al.* 2019; Zhang* et al.* 2019, 2020, 2021).

### Atomic force microscopy

Atomic force microscopy (AFM) studies the surface structure and properties of samples by detecting the extremely weak interatomic interaction between the sample surface and a miniature force-sensitive sensor (Binnig* et al.* 1986). In this approach, one end of a microcantilever that is extremely sensitive to weak forces is fixed, and the other end has a tiny needle tip (Fig. 1E). The needle tip contacts the sample surface lightly. During scanning, the microcantilever with the tip will undulate in the direction perpendicular to the sample surface (Binnig* et al.* 1986). Using the optical detection method, the position changes of the microcantilever corresponding to each scanning point can be measured, which reflects the sample surface morphology information (Binnig* et al.* 1986). High-speed (HS) AFM allows for the data acquired at high temporal resolution and has successfully revealed real-time conformational changes of SpCas9 (Table 1) (Shibata* et al.* 2017).

## MOLECULAR MECHANISMS OF SpCas9 REVEALED BY SINGLE-MOLECULE TECHNIQUES

SpCas9 is a large nuclease composed of 1,368 amino acids (Anders* et al.* 2014; Jiang* et al.* 2016b; Jinek* et al.* 2014; Nishimasu* et al.* 2014). SpCas9 undergoes a series of discrete DNA interrogation steps before cleavage. These steps are governed by critical DNA interactions and are often coupled with protein conformational changes (Anders* et al.* 2014; Jiang and Doudna 2017; Jiang* et al.* 2015, 2016b; Jinek* et al.* 2014; Nishimasu* et al.* 2014). Single-molecule techniques have offered unique experimental approaches to investigate different steps and aspects of the SpCas9 protein in the process of interrogating and cleaving DNA targets (Table 1). Below, we will highlight a few examples where single-molecule approaches have brought important insights into its molecular mechanisms.

### PAM search

Upon complexation with gRNA, the very first step in SpCas9 activity is to locate its DNA targets. Considering the vast amount of DNA in the cell, locating a 20–30-base pair protospacer could be time-consuming. How does the SpCas9–gRNA complex accelerate this process? Addressing this question necessitates the ability to simultaneously monitor both DNA and SpCas9–gRNA at the high spatiotemporal resolution, as DNA target recognition is an intrinsically transient protein–DNA interaction. DNA curtains and smFRET approaches are suitable for this task and have been applied to serve this purpose.

**Figure 2:**
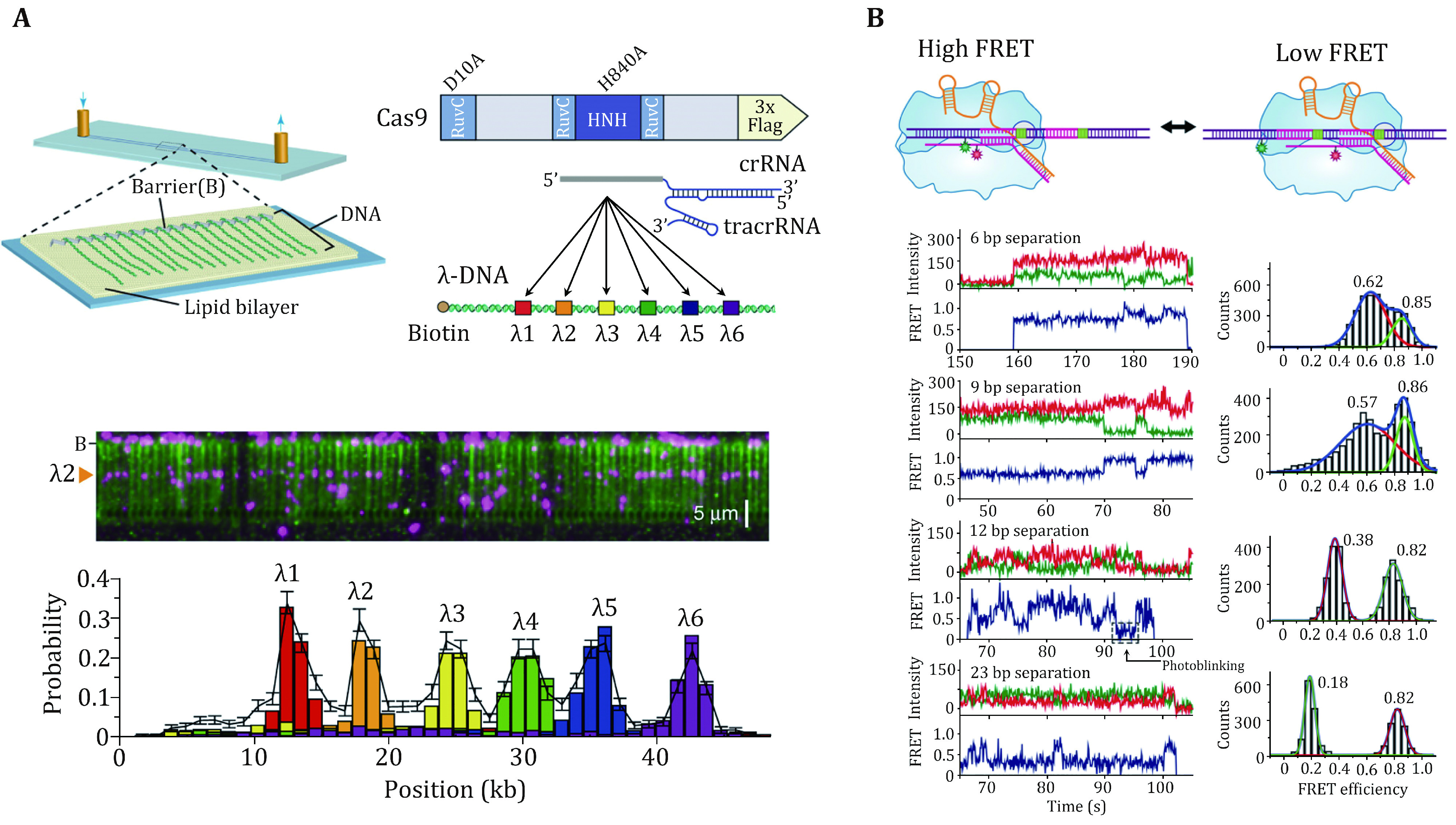
PAM search of SpCas9 revealed by single-molecule studies. **A** Schematic of the single-tethered DNA curtain for the SpCas9 PAM search assay. SpCas9-gRNA is designed to bind to six DNA target sites. SpCas9 binding sites are detected by DNA stained with YOYO1 (green) and SpCas9 labeled with QDs (magenta). **B** Schematic, smFRET traces, and histograms of SpCas9 binding to the PAMs at different locations, and the distance between PAMs is adjusted. The histograms show two FRET peaks corresponding to either of the target DNA sites. The high FRET peak remains constant across each histogram, while the low FRET peak moved towards the low FRET value as the distance between the targets increases. Adapted from Sternberg *et al.* (2014) and Globyte *et al.* (2019) with permissions

Sternberg *et al.* used a DNA curtain assay to visualize the target searching of a single SpCas9–gRNA complex in real time (Sternberg* et al.* 2014). The search process of the complex was monitored by imaging the YOYO1-stained λ-DNA molecule and quantum dot (QD)-labeled SpCas9 protein in complex with gRNA (Fig. 2A). They found that SpCas9–gRNA first searches for a dinucleotide PAM (5’-GG-3’) in a three-dimensional (3D) collision manner. The complex only transiently samples off-target sites, which is correlated with the PAM density and ignores sequences that are complementary to the gRNA but lack an adjacent PAM. These data suggest that SpCas9–gRNA begins the PAM search through a random collision with DNA. To accelerate the search, the complex reduces the time spent at non-PAM sites and interrogates only the PAM-flanking DNA for gRNA complementarity. In this assay, they observed no evidence of SpCas9–gRNA associating with targets by one-dimensional (1D) sliding/hopping. However, due to the diffraction limit of light microscopy (~250 nm), the 3D target search model may not be valid for a short length scale of nucleotides. To test that, Globyte *et al.* employed a smFRET experiment to examine the target searching process of a single SpCas9–gRNA complex in a smaller range (Fig. 2B) (Globyte* et al.* 2019). They immobilized a biotinylated SpCas9 complexed with Cy3-labelled gRNA on a PEG-coated quartz surface, followed by the injection of Cy5-labelled DNA into the reaction chamber. Using a series of DNA templates with a few PAMs placed at different distances, the experiment showed that weak PAM binding enables the complex to interrogate adjacent sequences with a range of ~20 bp in a facilitated 1D diffusion manner. These results suggest that the SpCas9–gRNA complex employs a combination of both 3D collision and 1D diffusion to locate its targets.

### Stable binding to a DNA target

**Figure 3:**
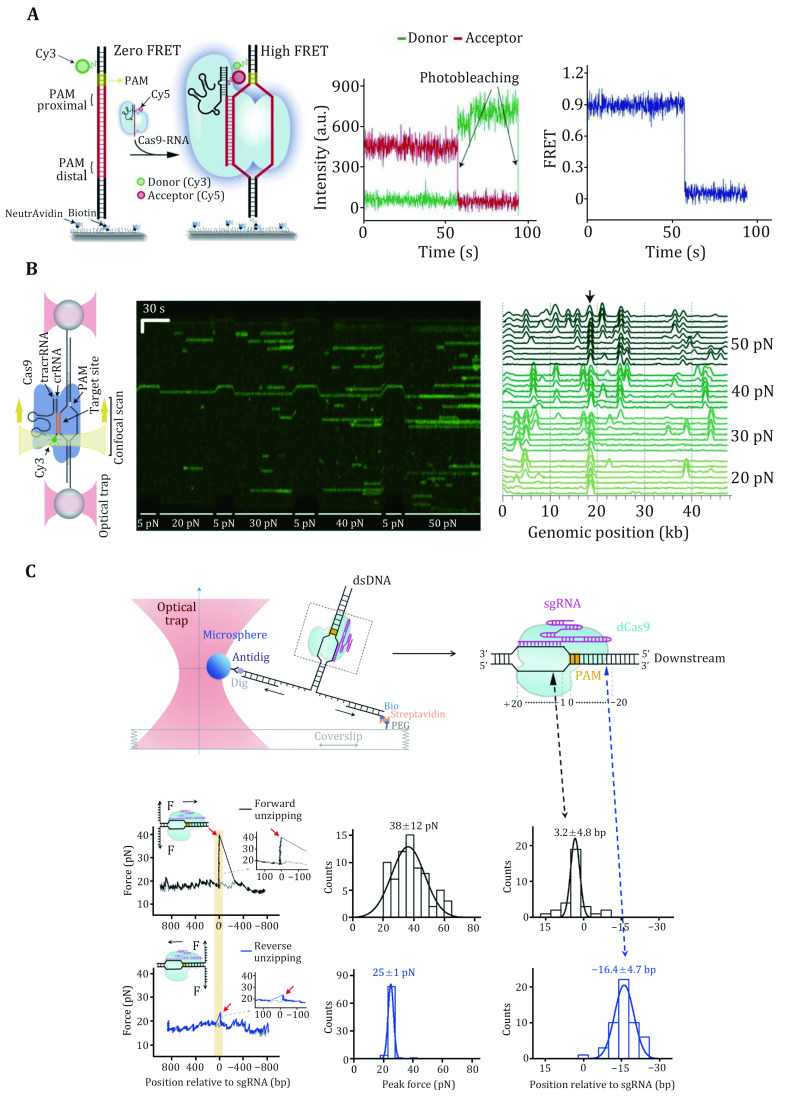
DNA binding of SpCas9 revealed by single-molecule studies. **A** Schematic and a representative trace of the smFRET assay for the SpCas9 binding target. **B** Kymograph and time-binned intensity histogram of force-stretched lambda DNA in the presence of 5–50 pN force, and off-target binding occurs once the force is higher than 20 pN. **C** Schematic of the single-molecule DNA unzipping experiment and the target DNA coordinate definition. Representative trace, disruption force histogram, and ternary interaction position histogram of forward (black) and reverse unzipping (blue) in the presence of SpCas9 bound to the target DNA. Adapted from Singh *et al.* (2016), Newton *et al.* (2019), and Zhang *et al.* (2019) with permissions

After PAM recognition, whether SpCas9–gRNA stably binds to or quickly dissociates from a DNA target relies on crRNA–DNA complementarity. To address how mismatches influence target recognition and DNA binding of SpCas9, Singh* et al.* designed smFRET assays to monitor real-time interactions between SpCas9–gRNA and DNA targets (Fig. 3A) (Singh* et al.* 2016). By labeling gRNA with Cy5 and DNA target with Cy3, they found that mismatches proximal to PAM greatly increase the SpCas9 dissociation rate (from <0.006 s^−1^ to >2 s^−1^), whereas PAM-distal mismatches still allow for the stable binding of the complex to DNA targets. Specifically, 9–10 PAM-proximal matches are sufficient for ultrastable SpCas9–gRNA binding. Moreover, as the dwell-time analysis shows two characteristic binding times, a two-step mechanism of Cas9–RNA binding involving PAM surveillance and RNA-DNA heteroduplex formation (see the next section) was proposed (Singh* et al.* 2016). In addition to PAM-distal mismatches, the fluorescence-combined optical tweezers and smFRET assays from the Rueda Laboratory revealed that DNA bubbles, driven by the mechanical forces on the DNA, could also boost stable binding of SpCas9 to off-targets, including mismatches in the PAM-proximal region (Fig. 3B) (Newton* et al.* 2019). Moreover, using magnetic tweezers, a similar enhanced off-target binding effect was also detected with supercoiled DNA substrates (Ivanov* et al.* 2020). These data suggest that bubbles and supercoiling in DNA substrates further increase the promiscuity of stable binding of SpCas9 to off-targets.

The stable binding of SpCas9–gRNA to DNA targets is achieved via direct interactions among the ternary complex. To quantitatively determine these essential interaction sites, Zhang *et al.* used an optical-tweezer-based DNA unzipping technique to probe them along the DNA sequence (Fig. 3C) (Zhang* et al.* 2019). In addition to a strong interaction located within the protospacer, they uncovered an unexpected interaction site located approximately 14 bp downstream of the PAM (post-PAM interaction), which is beyond the PAM and protospacer. Importantly, the loss or occupation of this relatively weak interaction site on DNA significantly attenuates SpCas9 binding. This site was recently verified to mediate DNA sampling and unwinding of SpCas9 (Yang* et al.* 2021; Zhang* et al.* 2021). Consistent with the smFRET data, they also found that a 9-bp PAM-proximal RNA–DNA complementarity was sufficient to support stable SpCas9 binding (Zhang* et al.* 2019). Interestingly, a transient interaction at approximately 15 bp relative to the PAM in the protospacer region among the ternary complex was frequently detected with imperfect RNA–DNA complementarity. This transient interaction was postulated to serve to mediate sensing of RNA–DNA complementarity (see the next section) and/or govern HNH domain mobility for cleavage (see the following section of DNA dissociation after cleavage).

### Protospacer DNA unwinding and R-loop formation

To examine crRNA–DNA complementarity, protospacer DNA must be unwound by SpCas9–gRNA in an ATP-independent manner. crRNA–DNA complementarity serves as a second layer of protection for SpCas9–gRNA against off-target binding, and DNA unwinding coupled with R-loop formation has proven to be a primary determinant of SpCas9 activity (Gong* et al.* 2018). In this process, the complex displaces the nontarget strand (NTS) and hybridizes the target strand (TS) of the protospacer with gRNA, allowing for the formation of a three-strand nucleic acid structure known as the R-loop (Jiang and Doudna 2017; Sternberg* et al.* 2014). *In vitro* biochemical assays have demonstrated that SpCas9–gRNA can tolerate PAM-distal mismatches, whereas PAM-proximal mismatches in the first 8–12 nucleotides are more deleterious (Sternberg* et al.* 2014). These findings suggest a unidirectional unwinding mechanism wherein DNA unwinding initiates from the PAM-proximal “seed” DNA sequence and propagates to the PAM-distal region.

**Figure 4:**
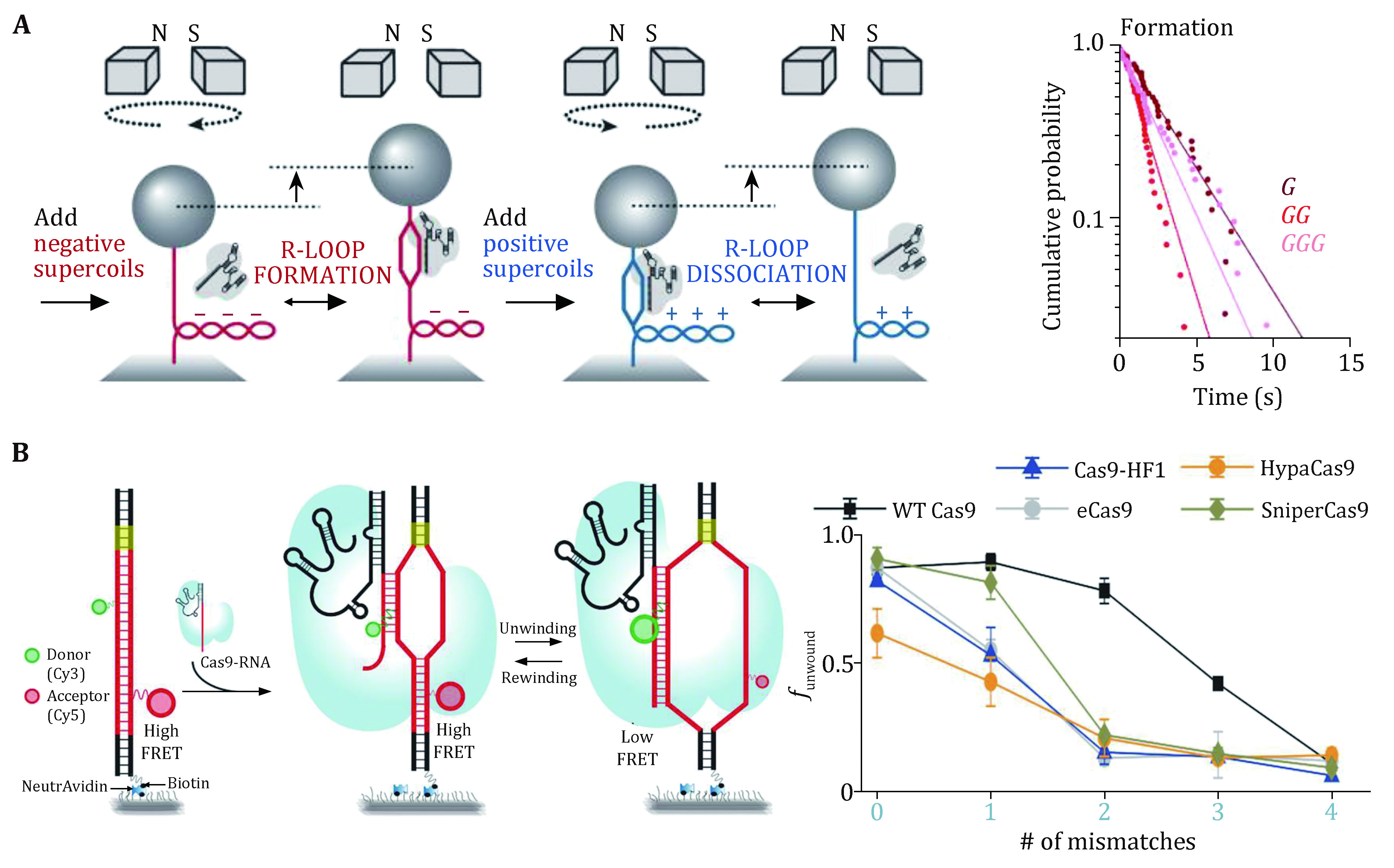
DNA unwinding and R-loop formation of SpCas9 revealed by single-molecule studies. **A** Schematic of the magnetic tweezers for the DNA unwinding assay. The inverse cumulative probability distribution of the time required for R-loop formation using different gRNAs. G, GG, and GGG represent the number of additional guanines at the 5’ end of gRNA. **B** Schematic of the smFRET assay for protospacer DNA unwinding by the SpCas9–gRNA complex. Statistical graph of the protospacer DNA unwinding probability with different numbers of DNA–RNA mismatches for the variety Cas9 mutants. Adapted from Mullally *et al.* (2020) and Okafor *et al.* (2019) with permissions

Magnetic tweezers were applied to study SpCas9 complexed with noncanonical gRNAs wherein 5’ modifications to gRNA were made (Fig. 4A) (Mullally* et al.* 2020). A 5’ addition of a 20-nt RNA hairpin to gRNA allowed for stable 9-bp R-loop formation, implying a discrete step in the unwinding of the protospacer DNA. Indeed, a magnetic-tweezer-based rotor bead tracking (RBT) study from the Bryant Laboratory exhibited a transient discrete intermediate in SpCas9–sgRNA-induced R-loop formation, consistent with RNA–DNA hybridization within an initial seed region (Ivanov* et al.* 2020).

Additional evidence for discrete DNA unwinding and R-loop formation came from a series of smFRET studies on the SpCas9 protein (Lim* et al.* 2016; Okafor* et al.* 2019; Singh* et al.* 2018; Zeng* et al.* 2018). In these studies, donor and acceptor dyes were separately placed on the TS and NTS within or around the protospacer region (or on the 5’ end of sgRNA), and protospacer DNA unwinding would result in the separation of the two dyes, thus a decrease in the FRET efficiency. A mid-FRET state was occasionally detected between the intact and fully unwound DNA states, suggesting a partially unwound DNA state (Lim* et al.* 2016; Zeng* et al.* 2018). Moreover, PAM-distal mismatches were found to impair DNA unwinding, and fully unwound protospacer DNA required at least 17-bp crRNA-DNA complementarity (Fig. 4B) (Singh* et al.* 2018). Engineered Cas9s, such as eCas9, Cas-HF1, HypaCas9, and SniperCas9, were proven to increase efficiency by partially depopulating the fully unwound state more readily with mismatches (Fig. 4B) (Okafor* et al.* 2019; Singh* et al.* 2018).

These single-molecule data support a model in which SpCas9-induced R-loop formation initiates from the PAM-proximal seed sequence and extends to the PAM-distal region. Before realizing the fully opened state, R-loop formation undergoes a partial intermediate state that is cleavage-incompetent. SpCas9–gRNA can remain stably bound to the DNA in this intermediate state (see the previous section “Stable binding to a DNA target”). Fully unwound protospacer DNA coupled with full R-loop formation possibly drives the docking of the HNH domain, thus licensing cleavage-competent SpCas9 (see the following section “DNA dissociation after cleavage”). Modifications of gRNA or the engineering of SpCas9 could rebalance the unwinding-rewinding equilibrium and make it stricter to reach the cleavage-competent state, thus minimizing off-target effects.

### Conformational rearrangements in SpCas9 domains

**Figure 5:**
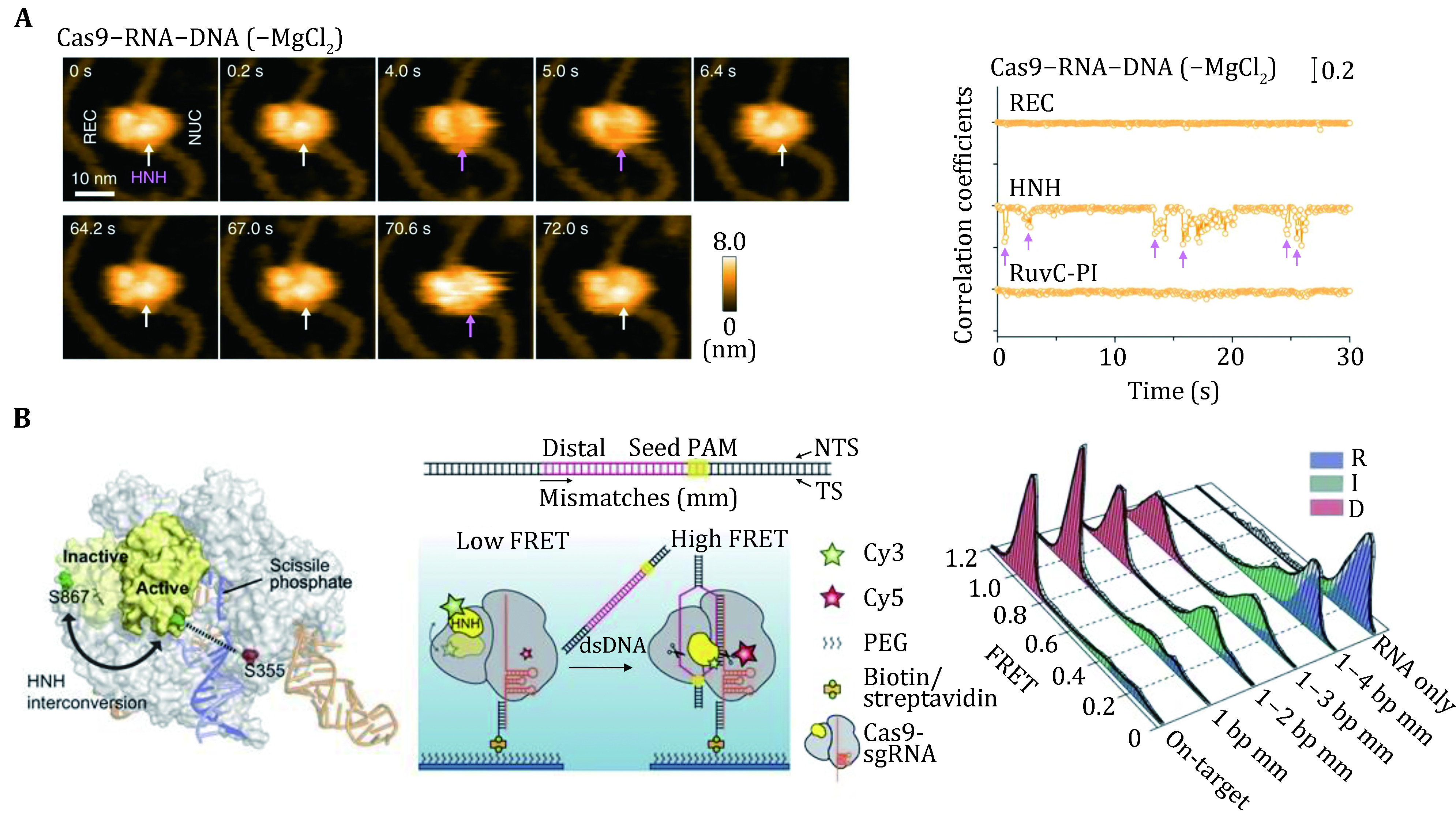
Conformational rearrangements of SpCas9 revealed by single-molecule studies. **A** Dynamic HS-AFM images of Cas9/RNA/DNA ternary in the absence of magnesium ions. The statistical graph of the correlation coefficient of each domain over time is shown on the right. The white arrows indicate the HNH domain, while the magenta arrows indicate the dynamic change of the HNH domain. The scale bar is 10 nm. **B** Schematic of different SpCas9 conformations (based on PDB 4ZT0 and 5F9R) and the smFRET assay for HNH domain dynamics. The steady-state histograms of SpCas9 with different numbers of DNA–RNA mismatches. Adapted from Shibata *et al.* (2017) and Dagdas *et al.* (2017) with permissions

SpCas9 is a multidomain DNA endonuclease. Structures of SpCas9 showed two distinct lobes, the alpha-helical recognition (REC) lobe and the nuclease lobe (NUS), as well as the more variable C-terminal domain (CTD) (Jinek* et al.* 2014). The NUC lobe contains the conserved HNH and split RuvC nuclease domains that are responsible for cleaving the TS and NTS, respectively. Crystal structures of apo, sgRNA-bound, sgRNA/DNA-bound SpCas9 have revealed the distinct conformational states of the protein, indicating that the protein must undergo conformational changes along its reaction pathway (Jiang and Doudna 2017). Indeed, a comparison of the structures of SpCas9–sgRNA complex and apo-SpCas9 reveals that gRNA can drive a substantial structural rearrangement of SpCas9 to realize a DNA recognition-competent conformation (Jiang* et al.* 2015; Jinek* et al.* 2014). Moreover, upon target binding and R-loop formation, SpCas9 undergoes a further conformation rearrangement that positions the HNH nuclease domain for the TS cleavage (Jiang* et al.* 2016b). A bulk FRET experiment first proved that the HNH domain samples a conformational equilibrium from an inactive state to an activated conformation (Sternberg* et al.* 2015), which was later confirmed by an HF-AFM study (Fig. 5A) (Shibata* et al.* 2017). smFRET studies have further examined the mobility of the HNH domain of SpCas9. Using SpCas9 variants labeled with Cy3 and Cy5 dyes, three groups identified an intermediate state of SpCas9 between the open and closed states, which represents the conformational checkpoint between DNA binding and cleavage (Fig. 5B) (Dagdas* et al.* 2017; Osuka* et al.* 2018; Yang* et al.* 2018). High-fidelity SpCas9 variants display slow transition rates to the active conformation, thus enhancing cleavage activity (Singh* et al.* 2018). Furthermore, smFRET studies revealed that a noncatalytic domain, REC3, governs HNH domain mobility. Recently, smFRET studies also demonstrated that the HNH domain of SpCas9 after cleavage was highly flexible (Wang* et al.* 2021).

### DNA dissociation after cleavage

**Figure 6:**
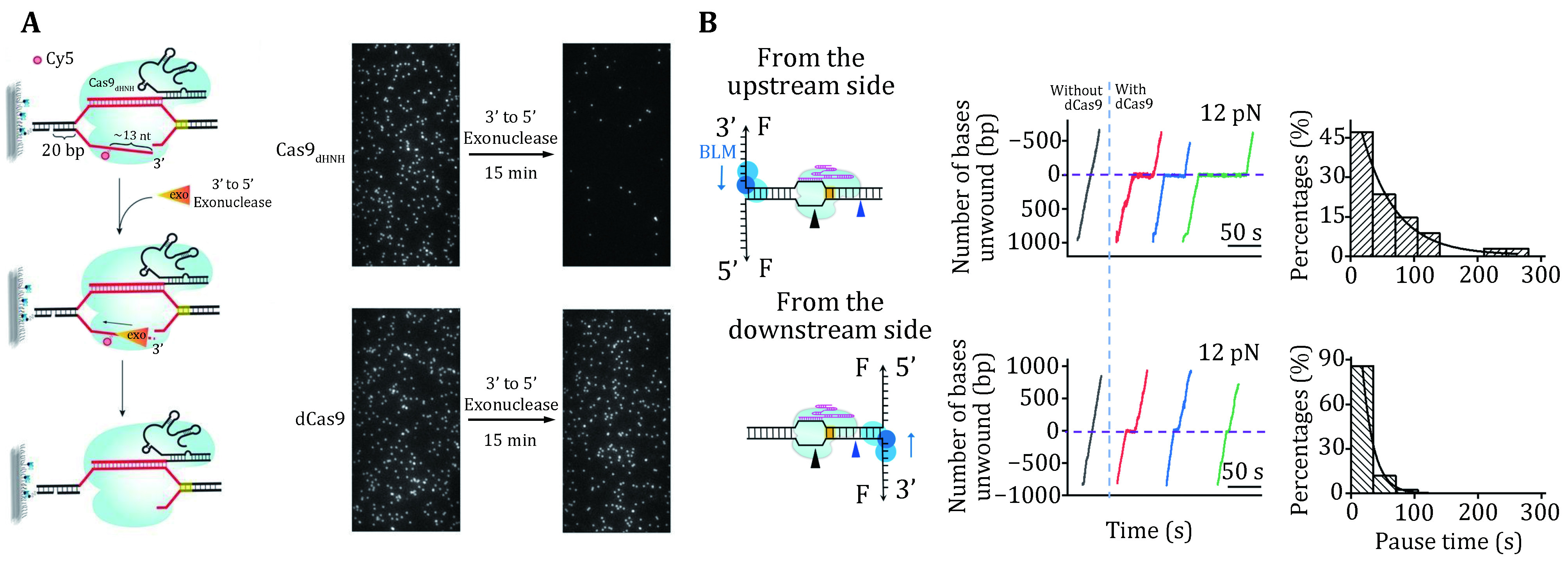
Single-molecule detection of SpCas9 postcatalytic dissociation. **A** Schematic of the fluorescence observation experiment for the 3’ flap NTS digestion. Representative images of the NTS digestion using SpCas9_HNH_ and SpCas9_dHNH_ before and after the Klenow fragments (the 3’ to 5’ exonuclease). **B** Schematic of the BLM helicase unwinding initiating from either the upstream (top) or downstream (bottom) side of the PAM. Representative traces show the number of unwound base pairs versus time under a constant force of 12 pN with or without dSpCas9. The histograms show the pause time of the BLM helicase at the expected dSpCas9 binding site. A single exponential fitting is used for these distributions. Adapted from Wang *et al.* (2021) and Zhang *et al.* (2019) with permissions

One distinguished characteristic of SpCas9 is its stable binding to the on-target site after cleavage. Both *in vitro* and *in vivo* experiments have demonstrated that SpCas9 remains tightly bound to both ends of the cleaved DNA for hours, thus acting as a single-turnover enzyme (Jones* et al.* 2017; Ma* et al.* 2016a; Sternberg* et al.* 2014; Zhang* et al.* 2019). At the single-molecule level, a DNA curtain assay provided evidence that SpCas9 bridges the double-strand break (DSB) ends for a long period unless there is a harsh treatment with 7 mol/L urea (Sternberg* et al.* 2014). An optical tweezer assay further verified that the ternary complex could sustain a rupture force of up to 40 pN (Newton* et al.* 2019). A fluorescence-based single-molecule digestion assay suggests that after cleavage by a SpCas9 nickase (SpCas9_dHNH_), the 3’ flap generated by the cleaved NTS is possibly exposed and can be digested by exonucleases (Fig. 6A) (Wang* et al.* 2021). Therefore, it is highly likely that different types of CRISPR proteins may employ distinct dissociation mechanisms after cleavage.

The long lifetime of the SpCas9–gRNA–DNA complex limits the efficient usage of each SpCas9 protein and impairs the repair of DSBs (Clarke* et al.* 2018). Increasing the slow off-rate of DNA-cleaved SpCas9 to DNA would be expected to improve its efficiency. Single-molecule experiments have demonstrated that DNA-based motor proteins, *in vitro*, could facilitate the dissociation of DNA-cleaved SpCas9 from DNA. Zhang *et al.* used optical tweezers to examine the consequence of encountering a BLM helicase with a DNA-bound dSpCas9 from both sides (Fig. 6B). They provided a proof of concept that, compared with the upstream side of the PAM, SpCas9 is more readily displaced from the downstream side of the PAM by BLM. These results highlight the importance of the post-PAM interaction in regulating DNA dissociation of SpCas9. Other motor proteins, such as Pif1, RNA polymerase, CMG helicase, and the histone chaperone FACT were also reported to be capable of dislodging DNA-bound SpCas9 (Clarke* et al.* 2018; Schauer* et al.* 2020; Vrtis* et al.* 2021; Wang* et al.* 2020).

## SUMMARY AND PERSPECTIVES

**Figure 7:**
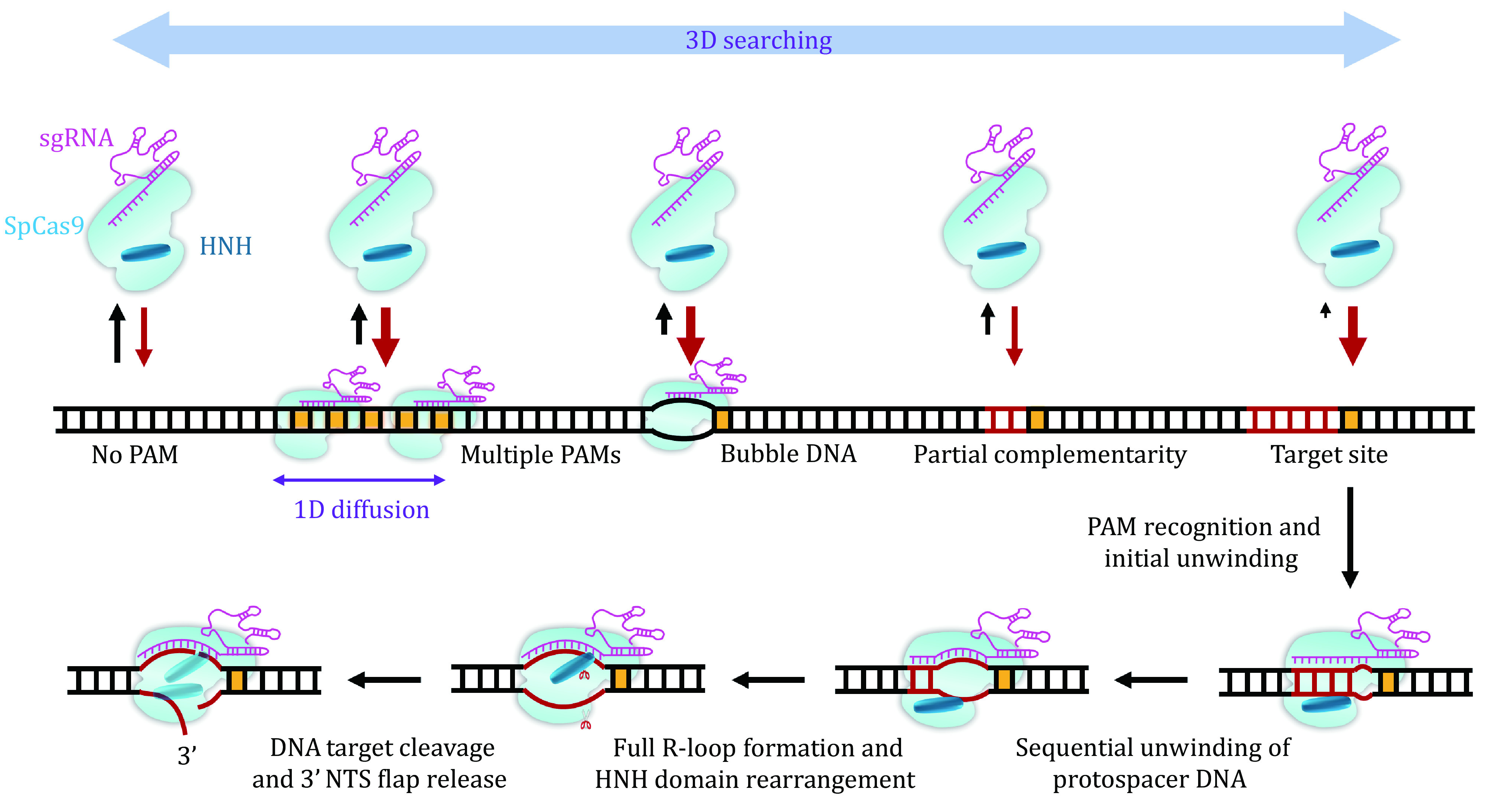
A dynamic model for the interplay between SpCas9 and DNA. The PAM search is carried out through random 3D collision, and 1D diffusion is performed near the PAM in a close region. DNA bubbles and crRNA–DNA complementarity promote the binding of SpCas9–sgRNA to the DNA. DNA binding by SpCas9–sgRNA induces the unwinding of the PAM-proximal protospacer DNA, giving rise to the formation of an RNA–DNA heteroduplex. The R-loop expansion propagates to the PAM-distal region. Driven by the complete formation of the R-loop, the HNH domain is repositioned to the cleavage site and the DNA is cleaved. SpCas9–sgRNA remains bound to the cleaved site wherein the cleaved 3’ flap NTS is first exposed

As evident from this review, single-molecule studies provide not only a fundamental understanding of Cas9 mechanisms but also a framework for rational design aiming at improving Cas9 efficiency and minimizing off-target effects. Based on these studies, a detailed dynamic picture of DNA interrogation and cleavage of SpCas9 has been generated (Fig. 7). Upon complexation with sgRNA, SpCas9 first uses a combination of 3D and 1D searching modes to target PAM. The binding of SpCas9 to the PAM initiates PAM-proximal protospacer DNA unwinding and an intermediate R-loop formation to examine crRNA–DNA complementarity. The first 8–10 bp crRNA–DNA matches are sufficient to support the stable binding of SpCas9–sgRNA to the target and will promote further unwinding of the protospacer DNA. Full R-loop formation will not be reached unless 17-nt or more protospacer DNA pairs with the crRNA. Complete annealing of crRNA and target DNA allows the HNH domain to reach a stable, active conformation for TS cleavage. Then, SpCas9 remains stably bound to both DSB ends wherein a cleaved NTS is exposed and can be accessed by other proteins. DNA-based motor proteins may promote final DNA dissociation from the target for future DNA repair. We believe that single-molecule techniques will continue to contribute to the CRISPR field.

## Conflict of interest

Qian Zhang, Ziting Chen and Bo Sun declare that they have no conflict of interest.
